# Thoracic Paravertebral Dedifferentiated Liposarcoma Masquerading as Spinal Tuberculosis: Case Report and Review of Literature

**DOI:** 10.1155/crra/4900055

**Published:** 2025-06-04

**Authors:** Liang Li, Jun-Long Pan, Cheng-Xin Yu, Peng Sun

**Affiliations:** ^1^Department of Radiology, The First College of Clinical Medical Science, China Three Gorges University and Yichang Central People's Hospital, Yichang, Hubei Province, China; ^2^Philips Healthcare, Beijing, China

**Keywords:** dedifferentiated liposarcoma, spinal tuberculosis, thoracic vertebrae

## Abstract

Primary poorly differentiated liposarcoma of the spine represents an exceptionally rare clinical entity. We present a 68-year-old patient with a poorly differentiated liposarcoma originating in the thoracic paraspinal region that was initially misdiagnosed as spinal tuberculosis. Although surgical decompression and subsequent biopsy confirmed the diagnosis of liposarcoma, the patient declined adjuvant chemotherapy and experienced disease recurrence within 2 months. This case underscores the critical consideration that differentiated spinal sarcomas may be radiologically indistinguishable from spinal tuberculosis, necessitating histopathological confirmation for accurate diagnosis.

## 1. Introduction

Spinal tuberculosis (Pott's disease) typically demonstrates characteristic imaging findings, including intervertebral disk space narrowing, adjacent vertebral body destruction, and paravertebral abscess formation [1]. We report a 68-year-old woman with a 3-year history of back pain whose thoracic spine CT and MRI findings initially suggested spinal tuberculosis. However, subsequent vertebroplasty and biopsy confirmed dedifferentiated liposarcoma (DDLS).

This case highlights that paravertebral DDLS can be radiologically indistinguishable from spinal tuberculosis. Importantly, the diagnosis of spinal tuberculosis should not rely solely on imaging features. While imaging findings—whether typical or atypical—may provide supportive evidence, definitive diagnosis requires histopathological confirmation through biopsy.

## 2. Case Report

A 68-year-old woman presented with a 3-year history of persistent back pain that was unresponsive to symptomatic management. Initial thoracic spine MRI at a referring hospital suggested T8–10 spinal tuberculosis (original imaging unavailable). She was subsequently transferred to our institution for comprehensive evaluation.

Physical examination revealed significant paraspinal tenderness with percussion pain. Laboratory investigations demonstrated normal inflammatory markers (ESR 16 mm/h, CRP 0.94 mg/L) and negative TB-Ab serology. Although these results did not definitively exclude spinal tuberculosis, the patient's inability to provide prior imaging and refusal of recommended radionuclide bone scanning necessitated additional CT and MRI studies.

**Figure 1:**
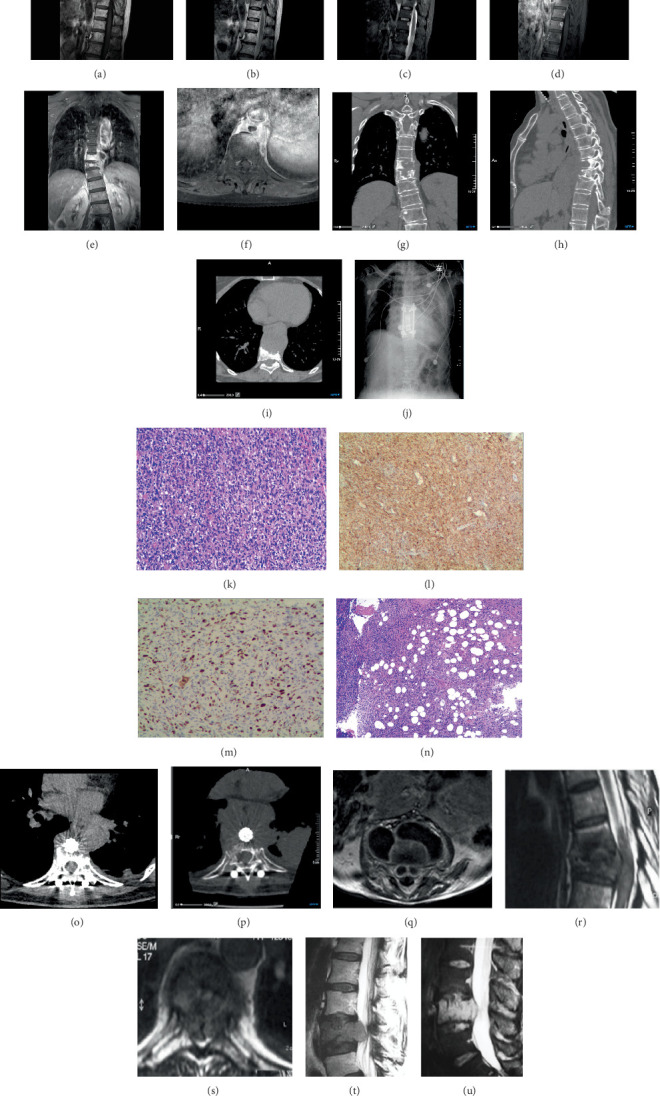
Sagittal (a) T1-weighted, (b) T2-weighted, (c) STIR, and (d) gadolinium-enhanced T1-weighted MRI images show destruction of the anterior portions of three adjacent vertebral bodies, along with localized narrowing of the intervertebral disc space. Gadolinium-enhanced T1-weighted (d) sagittal, (e) coronal, and (f) transverse MRIs demonstrate significant enhancement at the edges of the mass, with no enhancement in the center. (g) Sagittal, (h) coronal, and (i) transverse CT scans reveal bone destruction, vertebral collapse, narrowing of the intervertebral disc space, and paravertebral masses associated with the T8–9 vertebral body. (j) Postoperative bedside chest radiographs of the thoracic spine show internal fixation after the resection of the T8–10 vertebral body. (k) Histological findings reveal tumor cells that are spindle-shaped with heterogeneity, as shown by hematoxylin-eosin staining. Immunohistochemistry indicates positive expression of (l) CDK4 and (m) Ki-67 (60%), while (n) a few well-differentiated adipocytes and lipoblast-like cells are present in the tumor (hematoxylin-eosin staining). (o) A CT scan performed 2 months later showed new nodules in the paravertebral area and left pleura. (p) A CT scan conducted 6 months later indicated significant enlargement of the paravertebral and left pleural nodes. In a 5-year-old patient with pulmonary tuberculosis and gibbus, (q) a T1 axial image postgadolinium shows thin peripheral enhancement of paravertebral and intrathecal collections [1]. A sagittal MRI T2-weighted image, without fat saturation, demonstrates widening of the spinal canal, with partial preservation of fatty marrow in the plate of T7. (r) The vertebral bodies appear hypointense [2]. An axial MRI T2-weighted image shows bilateral narrowing of the spinal canal, with compression of the spinal cord by the posterolateral aspects of the tumor. (s) Surrounding tissues are not invaded [2]. MRI of the lumbar region in a sagittal slice shows (t) a hypointense signal in T1 and (u) a hyperintense signal in T2 [3].

The presentation of the three-dimensional computed tomography (3D-CT) scan images is consistent with spinal tuberculosis, highlighting anterior vertebral destruction resembling “worm-eaten” patterns, vertebral collapse, localized narrowing of the intervertebral disk space, and paravertebral masses associated with the T8–9 vertebral body (Figures 1g, 1h, and 1i). Further MRI enhancement was performed to clarify the nature of the paravertebral mass.

Contrast-enhanced magnetic resonance imaging (CE-MRI) of the thoracic spine revealed adjacent vertebral bone, a paravertebral mass with annular enhancement, and localized narrowing of the intervertebral disk space at the T8–9 vertebral bodies (Figures 1a, 1b, 1c, 1d, 1e, and 1f). The enhancement pattern of the paravertebral mass is considered indicative of a “paravertebral abscess.”

In conclusion, these findings initially suggested spinal tuberculosis and paravertebral abscess formation at T8–10. However, due to the patient's refusal of further investigations and the extensive involvement of the paravertebral abscess, we decided to proceed directly to surgery. An anterior approach to the thoracic spine was chosen for the surgical intervention. Intraoperatively, a hard paravertebral mass was observed at T8–9, characterized by caseous necrosis and fish-like tissues. The T8–10 vertebral body exhibited severe bone destruction filled with necrotic tissue, while the disk showed only edematous degeneration. Intraoperative frozen section examination of the lesion determined malignancy; therefore, we stripped and resected the tumor mass as thoroughly as possible. After resection of the T8–10 vertebral body portion, internal fixation was performed using a titanium cage implant loaded with autogenous bone graft debris, followed by posterior fusion with a pedicle screw implant. Postoperative radiographs confirmed the normal positioning of the internal fixation devices (Figure 1j).

Histopathology revealed that most tumor cells were arranged in a spindle shape; the tumor cell population was heterogeneous, characterized by numerous atypical nucleated cells accompanied by extensive degeneration and necrosis; and a few well-differentiated adipocytes and adipoblast-like cells were also present in the tumor (Figure 1k,n). Immunohistochemical analysis indicated the following results: SMA+, CD34−, S100−, HMB45−, PCK−, CD68+, CD117−, DOG-1−, Desmin−, H−, Cadherin−, Vimentin+, CDK4+, MDM2+, Ki-67 (60%), Myogenin−, MyoD1−, and ALK (1A4)− (Figure 1l,m). Combining these cellular features led to a final diagnosis of DDLS.

The patient's wound healed well, her vital signs remained stable, and her back pain improved. However, she consistently refused to undergo the recommended radiotherapy regimen. Follow-up at 2 months revealed new nodules in the paravertebral area and left pleura (Figure 1o), with significant enlargement of these nodular foci observed at the 6-month follow-up (Figure 1p). This was considered a recurrence of the paravertebral tumor and pleural metastasis, leading to subsequent loss to follow-up.

## 3. Discussion

Spinal tuberculosis was first reported in 1779. Although tuberculosis has become rare in developed countries, it remains prevalent in China [4]. Spinal tuberculosis accounts for approximately two-thirds of skeletal tuberculosis [5, 6]. It is estimated that more than 50% of patients with spinal tuberculosis lack evidence of pulmonary involvement [7], which explains the normal CT findings of the lungs in this patient. Our patient presented with back pain, a common but nonspecific clinical manifestation of early spinal tuberculosis [8]. In the absence of pathology, clinical manifestations and ancillary tests serve as the main diagnostic basis [9]. Imaging studies are invaluable for diagnosing spinal tuberculosis, revealing vertebral bone destruction, disk destruction, and paravertebral abscess formation [10, 11].

In studies of spinal tuberculosis, 65.5% of patients were found to have paravertebral abscesses [12]. The enhancement pattern of a paravertebral abscess is typically characterized by annular enhancement with smooth margins (Figure 1q), which did not apply to our case. Furthermore, the enhancement of tuberculous abscesses may appear diffuse and homogeneous, presenting as thin, thick, smooth, or nodular enhancements [1], which aligns with our case (Figures 1d, 1e, and 1f). Another factor contributing to our misdiagnosis was that classical spinal tuberculosis bone destruction predominantly occurs in the anterior column of adjacent vertebrae [10, 13], similar to the situation in this patient (Figures 1a, 1b, 1c, 1d, and 1h). Generally, disk disruption is a typical sign of spinal tuberculosis, but this patient exhibited only limited mild intervertebral space narrowing on MRI (Figure 1a,d). Additionally, reduced vascularization of intervertebral disks in elderly patients may obscure disk involvement, further complicating diagnosis [14], as was the case with our 68-year-old female patient.

In this case, CT and MRI imaging demonstrated typical features consistent with spinal tuberculosis, including destruction of the adjacent vertebral body, annular enhancement of the paravertebral mass, and localized narrowing of the intervertebral disk space. We maintained our diagnosis of spinal tuberculosis until histopathological examination revealed our misdiagnosis (Figures 1k, 1l, 1m, and 1n).

Liposarcoma is the second most common soft tissue sarcoma [15]. The recent 2020 update of the WHO classification of soft tissue sarcomas classified liposarcomas into five subtypes: well-differentiated, dedifferentiated, myxoid, pleomorphic, and myxoid pleomorphic [16]. DDLS was first introduced by Evans et al. in 1979 as a high-grade, nonlipogenic sarcoma [17]. It is most commonly seen in the retroperitoneum but can occur at other sites in the body [18]. The spine is a rare site for such tumors, and we have encountered only 12 case reports of imaging presentations that show vertebral bone destruction or disk involvement [2, 3, 19] (Figures 1r, 1s, 1t, and 1u). We found no prior reports of vertebral liposarcoma accompanied by paravertebral abscess formation, which contributed to our initial misdiagnosis. The imaging findings strongly suggest a diagnosis of spinal tuberculosis, although pathology ultimately confirmed DDLS (Figures 1k, 1l, 1m, and 1n).

Determining whether the tumor originated in the paravertebral area or the vertebral body was challenging. We considered the paravertebral mass as the primary lesion, as it encircled the artery, and the vertebral bone destruction was primarily anterior. Consequently, we diagnosed this case as retroperitoneal DDLS.

The differential diagnosis between spinal tuberculosis and tumors can vary across different regions due to the differing incidence of these diseases and may even be misdiagnosed or overlooked due to inertia [10]. The typical imaging presentation and clinical signs strongly suggest tuberculous spondylitis, particularly in developing countries where tuberculosis remains prevalent. However, the pseudo “paravertebral abscess” presented in this case may represent an unusual manifestation of a poorly differentiated liposarcoma invading the spine and is likely to be misdiagnosed due to the similarities in imaging. Histological and immunohistochemical examinations are essential for accurate final diagnoses and further treatment decisions.

## Data Availability

The authors confirm that the data supporting the findings of this study are available within the article.
